# *Paris polyphylla* Sm. Induces Reactive Oxygen Species and Caspase 3-Mediated Apoptosis in Colorectal Cancer Cells In Vitro and Potentiates the Therapeutic Significance of Fluorouracil and Cisplatin

**DOI:** 10.3390/plants12071446

**Published:** 2023-03-25

**Authors:** Vimi Kshetrimayum, Rameshwari Heisnam, Ojit Singh Keithellakpam, Pullapanthula Radhakrishnanand, Sai Jyothi Akula, Pulok K. Mukherjee, Nanaocha Sharma

**Affiliations:** 1 Microbial Resources Division, Institute of Bioresources and Sustainable Development Takyelpat, Imphal 795001, India; 2 School of Biotechnology Kalinga Institute of Industrial Technology (KIIT), Deemed to be University, Bhubaneshwar 751024, India; 3 Department of Pharmaceutical Analysis, National Institute of Pharmaceutical Education and Research, Guwahati 781101, India

**Keywords:** apoptosis, cytotoxicity, steroidal saponins, synergistic

## Abstract

*Paris polyphylla* Sm. (Melanthiaceae) is an essential, vulnerable herb with a wide range of traditional applications ranging from fever to cancer in various communities. The use of *P. polyphylla* in India is limited to traditional healers. Here, we demonstrated that *P. polyphylla* extract (PPE) has good phenol, flavonoid, saponin, and steroidal saponin content and anti-oxidant activity with IC_50_ 35.12 ± 6.1 μg/mL in DPPH and 19.69 ± 6.7 μg/mL in ABTS. Furthermore, PPE induces cytotoxicity in HCT-116 with IC_50_ 8.72 ± 0.71 μg/mL without significant cytotoxicity inthe normal human colon epithelial cell line, CCD 841 CoN. PPE inhibits the metastatic property and induces apoptosis in HCT-116, as measured by Annexin V/PI, by increasing the production of reactive oxygen species (ROS) and caspase 3 activation. PPE acts synergistically with 5FU and cisplatin in HCT-116 and potentiates their therapeutic significance. Steroidal saponins with anticancer activities were detected in PPE by HR-LCMS. The present study demonstrated that PPE induces apoptosis by increasing ROS and activating caspase 3, which was attributed to steroidal saponins. PPE can be used as a potential natural remedy for colon cancer.

## 1. Introduction

*P. polyphylla*, commonly known as love apple in English, is a perennial rhizomatous herb that belongs to the Melanthiaceae family. *P. polyphylla* inhabits various temperate forests, ranging from the Himalayas to Western China and prefers an elevation between 1800 and 3300 m above sea level [1]. The best conditions for this species to thrive are in old-growth forests with more than 80% canopy cover and moist and shady conditions [2,3].The rhizomatous herb is distributed in Bangladesh, Bhutan, China, India, Laos, Myanmar, Nepal, Taiwan, Thailand, and Vietnam. In India, the herb has been documented in Arunachal Pradesh, Assam, Himachal Pradesh, Jammu and Kashmir, Manipur, Meghalaya, Mizoram, Nagaland, Sikkim, and Uttarakhand [1,4]. The ethnobotanical uses of *P. polyphylla* in India and Nepal include treatment of fever, diarrhoea, dysentery, and other gastrointestinal ailments, elimination of helminthes, or application as antidotes [5,6,7]. In Manipur, the Tangkhul tribes of the Ukhrul district consume the raw rhizomes of *P. polyphylla* to treat stomach ulcers [8]. Traditional Chinese Medicine (TCM) uses the rhizome of *Paris*(Chong Lou) as an integral part of its treatments, which include abnormal uterine bleeding, cancer, snake bites, skin disease, etc. [9]. It is also a significant primary ingredient for ‘Yunnan Baiyao’ and ‘Gong Xue Ning’ capsules. Many Chinese patented medicines contain Rhizome Paridis as an ingredient, including “Lou lianjiaonang” and “Jinfukangkoufu ye”, which are used as adjuvant therapies to enhance the benefits of cancer treatment [10]. The anticancer property of *P. polyphylla* has been reported for breast [11,12], liver [9], lungs [13], cervical [14], gastric [15], ovarian [16], and esophageal [17] cancers, etc. In addition to its anticancer property, *P. polyphylla* has also been shown to have anti-leishmanial [18], immunostimulant [19], hemostatic [4], anthelmintic [20], and antibacterial properties [21]. *Paris* saponins, or steroidal saponins, are the main bioactive chemical constituents in this plant, accounting for more than 80% of the total compounds. Steroid saponins, such as polyphyllin D, Diosgenin, and Paris saponins I, II, VI, VII, and H exhibit anticancer activity comparable to synthetic anticancer medicines [22].

Colorectal cancer (CRC) is of concern, as it is often diagnosed at an advanced stage, and the chances of survival for patients in stage IV are less than 10%. It is important to note that colorectal cancer is not sensitive to chemotherapy and immunotherapy, which is generally the cause of complications [23]. Fluorouracil (5FU) is the primary chemotherapeutic drug for treating colorectal cancer, although its benefit is momentarily associated with recurrence and side effects [24]. Even though the efficacy of 5FU has increased owing to adjuvant therapy, new approaches to enhance the activity of 5FU in CRC treatment are required [25]. In addition, the chemotherapy drug cisplatin often results in resistance in CRC patients [26]. This necessitates exploring a potent alternative natural compound with reduced side effects. The anti-CRC activity of *P. polyphylla* from China has been reported [27,28].

The northeastern region of India reported the highest incidence of colorectal cancer [29]. However, the activity of *P. polyphylla* from India for CRC has not been investigated. IUCN (TheInternational Union for Conservation of Nature) has classified *P. polyphylla* as “vulnerable” due to a decline in the wild population caused by overexploitation, habitat degradation, illegal collection for trade, and traditional usage [30]. There have been reports that the rhizome of this plant has been illegally exported to China via the Indo-Myanmar border in Manipur [31]. Therefore, people in Manipur are unaware of the benefits and therapeutic significance of *P. polyphylla*. The present study evaluates the total phenolic, flavonoid, saponin, and steroidal saponin content and anti-oxidant and anti-CRC properties of *P. polyphylla* collected from the Ukhrul district of Manipur, India. *P. polyphylla* extract (PPE) was also evaluated to determine its efficacy in combination with 5FU and cisplatin to improve its therapeutic potential. HR-LCMS was used to identify the bioactive compounds in PPE. The present study will uplift the therapeutic significance of *P. polyphylla* found in Manipur, India.

## 2. Results

### 2.1. Extraction Yield

The yield of the 20 g ethanol (70%) extraction of *P. polyphylla* was 4.6%.

### 2.2. Total Phenolic and Flavonoid Contents of PPE

Phenols and flavonoids are plant secondary metabolites essential for plants; they are valuable for human health and used as nutraceuticals to prevent several infectious, degenerative diseases, and allergies as well asfor application in cosmetic formulations for anti-ageing purposes [32,33]. Upon quantification of PPE, total phenolic content (TPC) was 34.4 ± 0.06 mg gallic acid equivalent (GAE)/g, and total flavonoid content (TFC) was 41.6 ± 0.09 mg quercetin (QE)/g.

### 2.3. Total Saponins and Steroidal Saponin Contents of PPE

Saponins constitute a vital ingredient of many traditional and folk medicines, with a wide range of pharmaceutical properties [34]. Pharmaceutical properties of saponins, such asanticancer [35], hemolytic activities [36], adjuvant immunologicalactivities [37], etc., have been reported.The PPE total saponin and steroidal saponin content was 270.92 ± 0.19 mg Diosgenin equivalent (DE)/g and 14.5 ± 0.02 mg Diosgenin equivalent (DE)/g, respectively.

### 2.4. Anti-Oxidant Activity

**Figure 1:**
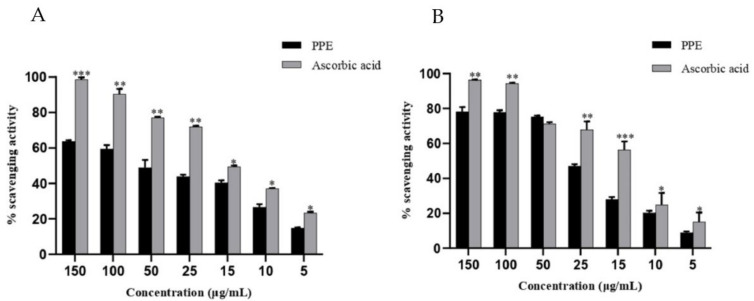
Anti-radical activity of PPE and standard ascorbic acid in various concentrations 5–150 μg/mL as determined by using (**A**) DPPH and (**B**) ABTS.The values indicate ± SD (*n* = 3); * *p* < 0.05, ** *p* < 0.01, and *** *p* < 0.001 vs. PPE.

The free-radical-scavenging activity of PPE was evaluated by DPPH and ABTS assays, and its activity was compared with that of standard ascorbic acid. PPE showed dose-dependent anti-oxidant activity comparable to ascorbic acid. At the highest concentration of 150 μg/mL, PPE showed 63% free-radical-quenching activity in DPPH, while in ABTS, it was 78% (Figure 1). The IC_50_ of PPE in DPPH was 35.12 ± 6.1 μg/mL, and ABTS was 19.69 ± 6.7 μg/mL (Table 1).

### 2.5. PPE Is Cytotoxic in HT-29, HCT-15, and HCT-116 but Less Cytotoxic in CCD 841 CoN

**Figure 2:**
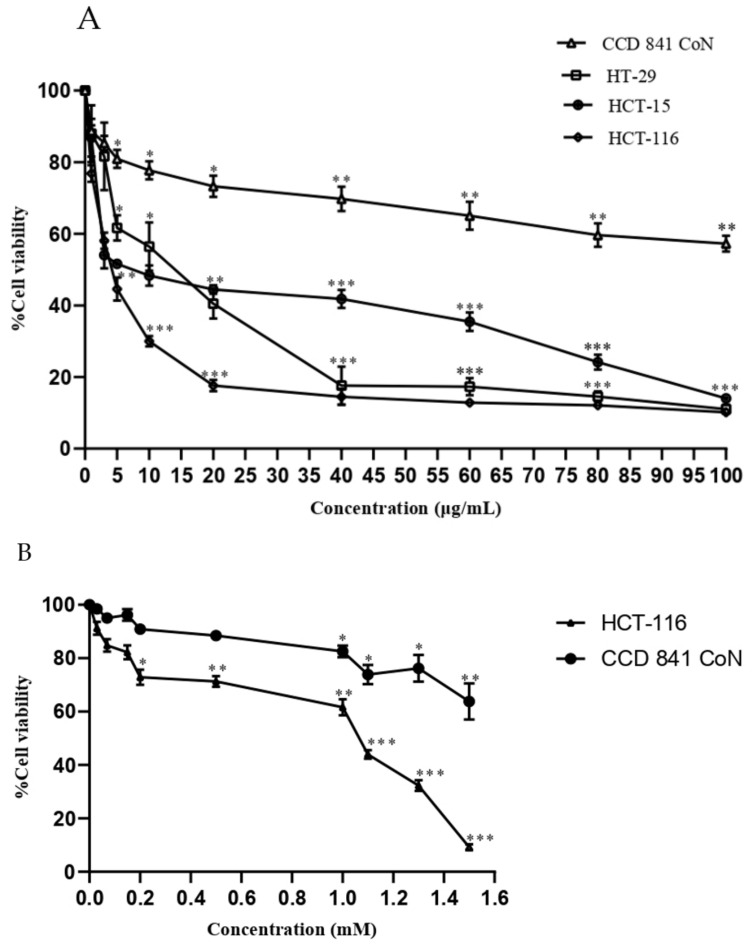
HT-29, HCT-15, HCT-116, and CCD 841 CoN cells were seeded in 96-well plates and treated with varying concentrations of PPE (1–100 μg/mL) and 5FU (0.03–1.5 mM) for 24 h. Its cytotoxic activity was evaluated by MTT (**A**) Effect of PPE on the viability of HT-29, HCT-15, HCT-116, and CCD 841 CoN; (**B**) Effect of 5FU on the viability of HCT-116 and CCD 841 CoN.The values indicate ± SD (*n* = 3); * *p* < 0.05, ** *p* < 0.01, and *** *p* < 0.001 vs. control.

The PPE cytotoxicity was determined in HT-29, HCT-15, HCT-116, and CCD 841 CoN by MTT (3-[4,5-dimethylthiazol-2-yl]-2,5-diphenyl tetrazolium bromide) assay for 24 h. PPE was cytotoxic in all the CRC cell lines tested. PPE drastically decreased the viability of HCT-116 with IC_50_ at 8.72 ± 0.71 μg/mL as compared with HT-29 and HCT-15 with IC_50_ at 12.94 ± 1.8 μg/mL and 10 ± 0.5 μg/mL, respectively. At 24 h of treatment, the viability of HT-29, HCT-15, and HCT-116 at 100 μg/mL was 14.06%, 11.01%, and 3.7%, respectively, showing that PPE had extremely high cytotoxicity in HCT-116 (Figure 2A). The viability of CCD 841 CoN upon treatment with PPE at 100–20 μg/mL was 65–75%, while at 10–1 μg/mL, the cell viability was 79–95% (Figure 2A). For 5FU at 1.5–0.5 mM, the cell viability of HCT-116 was 9–71%, while at 0.2–0.03 mM, the cell viability was 72–91% (Figure 2B). The viability of the normal human colon epithelial cell line, CCD 841 CoN, was higher compared with the cancer cell lines upon treatment with PPE and 5FU. Table 2 represents the respective IC_50_values of colon cancer and normal human colon epithelial cells treated with PPE.

### 2.6. PPE Reduces Colony Formation in HCT-116

**Figure 3:**
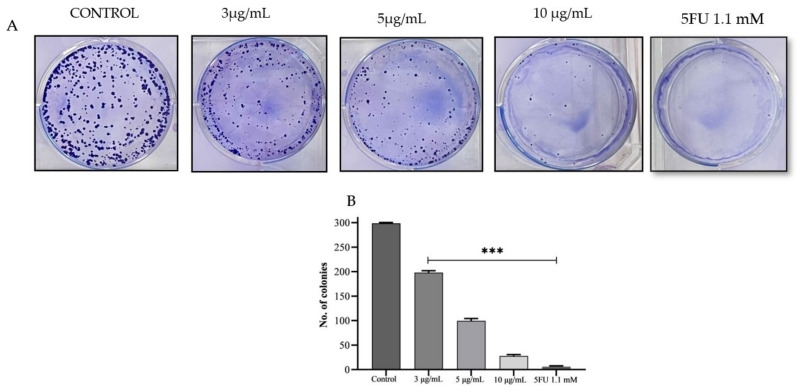
HCT-116 cells with density 600–700 were seeded and treated with PPE (3–10 μg/mL) and 5FU (1.1 mM). After 24 h, the extract was removed and replaced with fresh media, and the colony-forming capability was observed for 10 days and stained with crystal violet. (**A**) A dose-dependent decrease in the number of colonies; (**B**) Graphical representation of the number of colonies of HCT-116 treated with increasing doses of PPE. The values indicate ± SD (*n* = 3); *** *p* < 0.001 vs. control.

PPE showed the highest cytotoxicity in HCT-116. Further, the activity towards colony formation in HCT-116 was evaluated (Figure 3). At the minimum concentration of PPE 3 μg/mL, 65% colony was formed compared with the control. At 10 μg/mL, % of colony formation was 9% compared with the control. 5FU treatment showed significantly less colony.

### 2.7. PPE Inhibits HCT-116 Cells Migration

**Figure 4:**
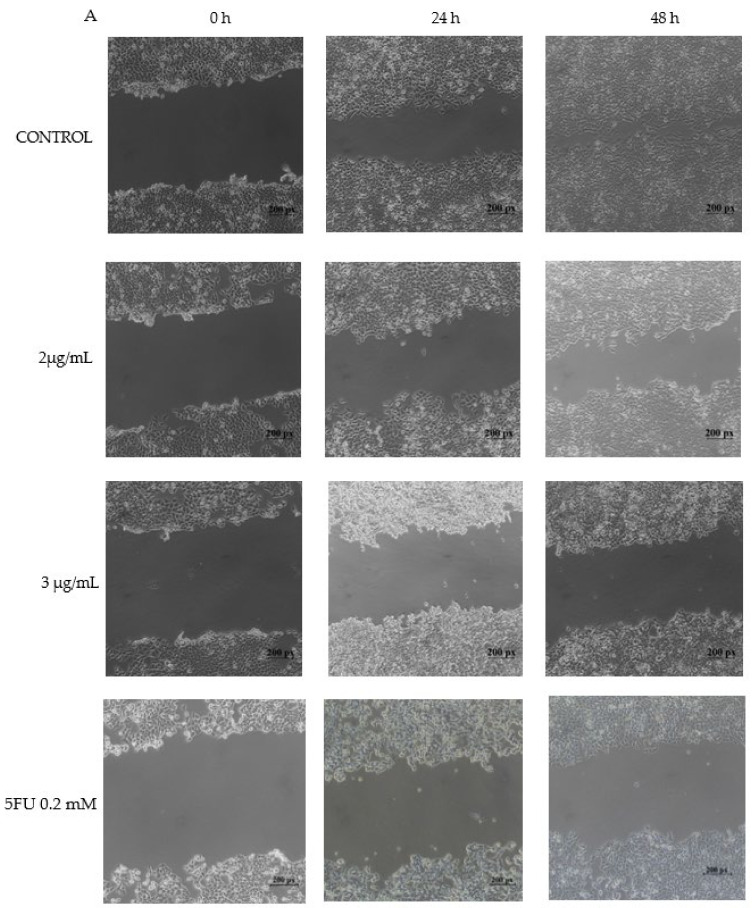

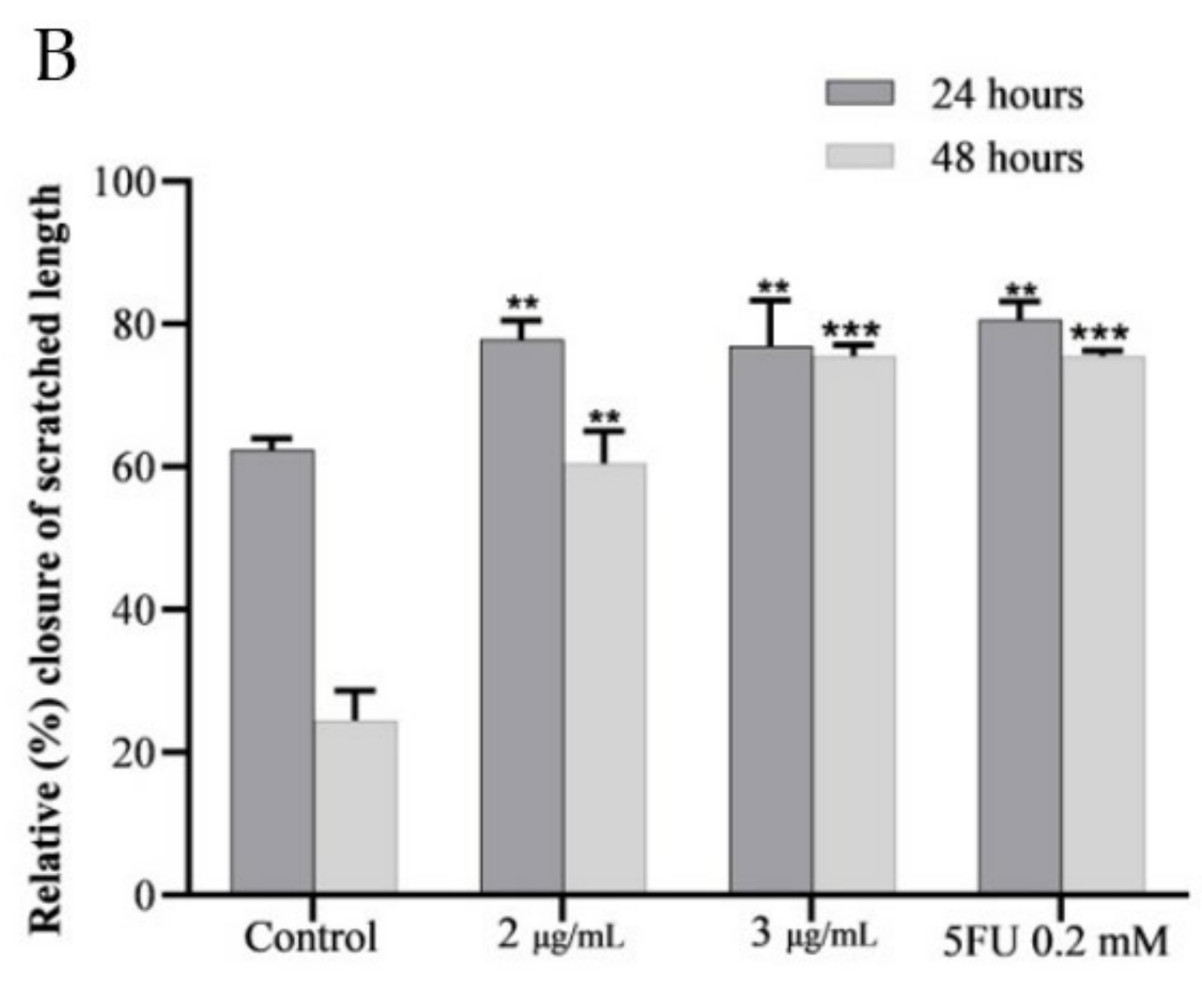
HCT 116 cells were seeded in 6-well plates and allowed to grow until 80–90% confluence, then scratched with a sterile 200 μL tip to create a wound and further treated with PPE (2 μg/mL and 3 μg/mL) or 5FU (0.2 mM). (**A**) Images of the scratched wound of the control, PPE-, and 5FU-treated recorded at 0 h, 24 h, and 48 h; (**B**) Relative closure length of the scratched wound as compared with control.The values indicate ± SD (*n* = 3); ** *p* < 0.01 vs. control and *** *p* < 0.001 vs. control.

HCT-116 scratch wound assay was performed to determine the inhibition potential of PPE in cell migration. HCT-116 was treated with a non-cytotoxic concentration of PPE (2 μg/mL and 3 μg/mL) and 5FU (0.2 mM). After 48 h, the control cells migrated towards the scratched area, whereas the PPE treated cells migrated to a lesser degree. PPE significantly halts the migration of HCT-116 cells in a dose- and time-dependent manner (Figure 4) comparable to 5FU.

### 2.8. Dual Role of PPE and Ascorbic Acid as an Anti-Oxidant and Prooxidant in HCT 116

**Figure 5:**
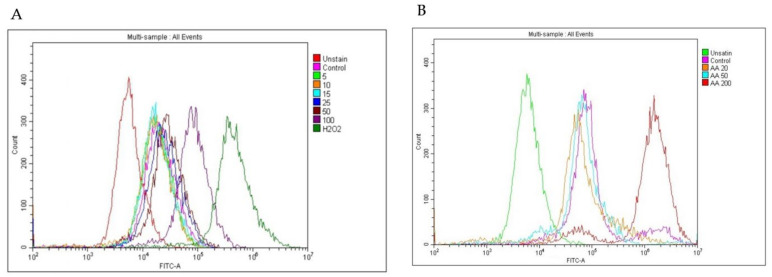
HCT-116 cells were treated with PPE (5–100 μg/mL) and H_2_O_2_ (500 μM) for 6 h and AA (20 μg/mL, 50 μg/mL, and 200 μg/mL) for 24 h. The cells were incubated with H_2_DCFDA for 30 min at 37 °C, and the fluorescent intensity was measured with a flow cytometer (BD Canto II). (**A**) DCF intensity of HCT-116 treated with PPE (5–100 μg/mL) and H_2_O_2_ (500 μM); (**B**) DCF intensity of HCT-116 treated with AA (20 μg/mL, 50 μg/mL, and 200 μg/mL).

The intensity of 2′,7′-dichlorofluorescein (DCF) was measured by flow cytometry to determine whether PPE treatment induced intracellular ROS generation in HCT-116. The prooxidant and anti-oxidant activity of ascorbic acid (AA) at high and low doses was also studied in HCT-116. PPE at 5 μg/mL, 10 μg/mL, and 15 μg/mL showed lower DCF intensity as compared with the control, while at 25 μg/mL, 50 μg/mL, and 100 μg/mL, the DCF intensity increased as compared with control (Figure 5A). Positive control H_2_O_2_ (500 μM) also showed an increase in DCF intensity (Figure 5A).Similarly, AA at 20 μg/mL and 50 μg/mL showed lower DCF intensity as compared with control, while at 200 μg/mL, the DCF intensity increased as compared with control (Figure 5B). This shows that PPE and AA act as anti-oxidants at low dose and as prooxidants at high dose in HCT-116.

### 2.9. PPE Induces Apoptosis in HCT-116

**Figure 6:**
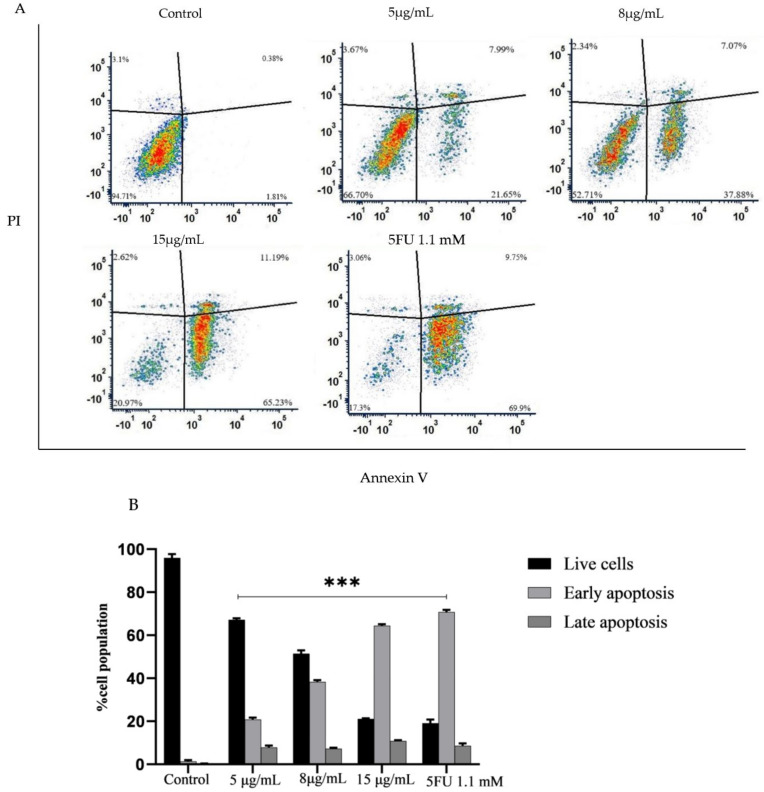
HCT-116 cells were treated with PPE (515 μg/mL) and 5FU (1.1mM) for 24 h, and a percentage of apoptosis was detected by staining with Annexin/PI. (**A**) Scatter plot of Annexin V and PI on X and Y axis, respectively, (**B**) Graphical representation of the live cells, early apoptotic and late apoptotic cells.The values indicate ± SD (*n* = 3); *** *p* < 0.001 vs. control.

To determine whether the decrease in cell viability observed after treatment with PPE was caused by apoptosis, HCT-116 cells were stained with Annexin V/propidium iodide (PI). Annexin V stains externalized phosphatidylserine (PS) released during early and late apoptosis. In necrotic or late apoptotic cells, PI stains the cells. Treatment with PPE (5–15 μg/mL) and 5FU (1.1 mM) for 24 h in HCT-116 cells ledto an increase in the percentage of apoptotic cells in a dose-dependent manner compared with the untreated control (Figure 6). PPE induced apoptosis in HCT-116 comparable to 5FU.

### 2.10. PPE Activates Caspase 3 in HCT-116

**Figure 7:**
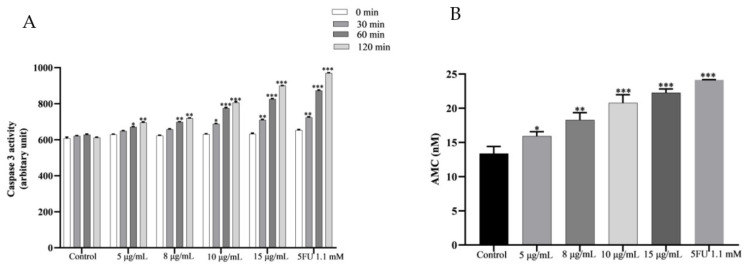
HCT-116 cells treated with PPE (5–15 μg/mL) and 5FU 1.1 mM for 24 h were assayed for caspase 3 activity. (**A**) Increase in caspase 3 activity with time at increasing PPE concentration comparable to 5FU; (**B**) AMC concentrations at increasing PPE concentrations or with 5FU 1.1 mM (120 min). The values indicate ± SD (*n* = 3); * *p* < 0.05, ** *p* < 0.01, and *** *p* < 0.001 vs. control.

Cells undergo apoptosis when caspase- or cysteine-dependent aspartate-specific proteases are activated [38]. Using the EnzChek^®^ Caspase-3 Assay Kit, PPE caspase 3 activity was evaluated by activatingZ-DEVD–AMC(7-amino-4-methylcoumarin-derived)-specific substrate in HCT-116. With the increase in the concentration of PPE, the caspase 3 activity increased gradually from 5–15 μg/mL with time (30 to 120 min) (Figure 7A). 5FU (1.1 mM) also showed an increase in caspase 3 activity with respect to time. The amount of AMC released in the reaction also escalated with the increase in PPE concentration (Figure 7B). Caspase 3 activation in HCT-116 by PPE was comparable to 5FU.

### 2.11. PPE Infusion with 5FU and Cisplatin

**Figure 8:**
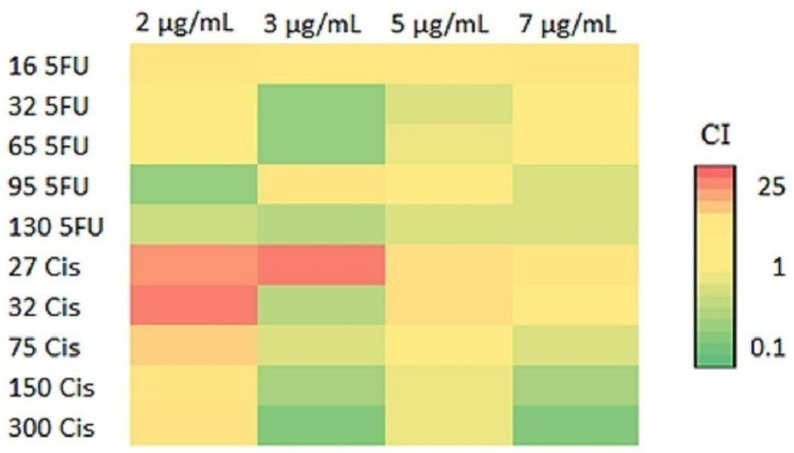
Heatmap for predicted drug combination effects of PPE with 5FU and cisplatin. The predicted CI values from the most synergistic to the most antagonistic pair arecolored from green to red.

The effect of synchronous treatment with sub-lethal doses of PPE with 5FU and cisplatin in HCT-116 was determined by MTT assay. Fortydifferent combinations of PPE with 5FU and cisplatin were evaluated. The combination index (CI) and cell viability of all the combinations studied are reported in Table 3. A heatmap representing the predicted drug combination effects of PPE with 5FU and cisplatin is shown in Figure 8, where 16 μg/mL of 5FU and 27 μg/mLof cisplatin showedantagonist combination with 2, 3, 5, and 7 μg/mL of PPE. Meanwhile, 130 μg/mL of 5FU showed synergistic effect with all the studied combinations of PPE. In addition, 300 μg/mL of cisplatin also showed synergistic combination with 3, 5, and 7 μg/mL of PPE.

### 2.12. Phytochemical Analysis of PPE

**Figure 9:**
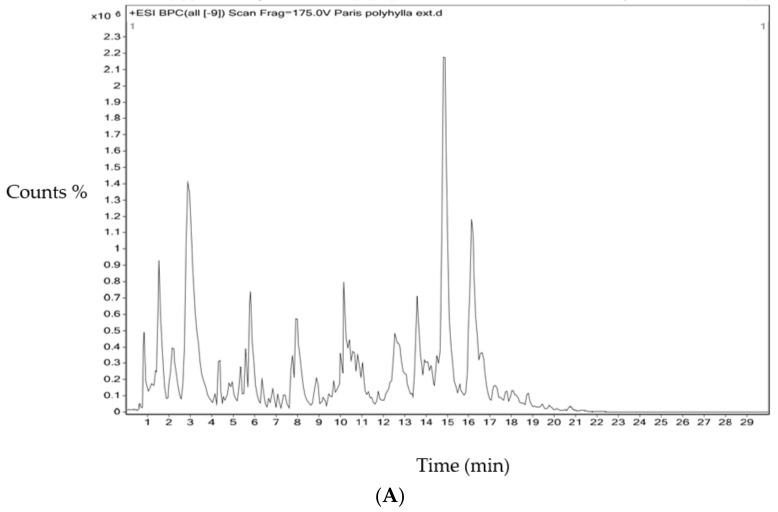

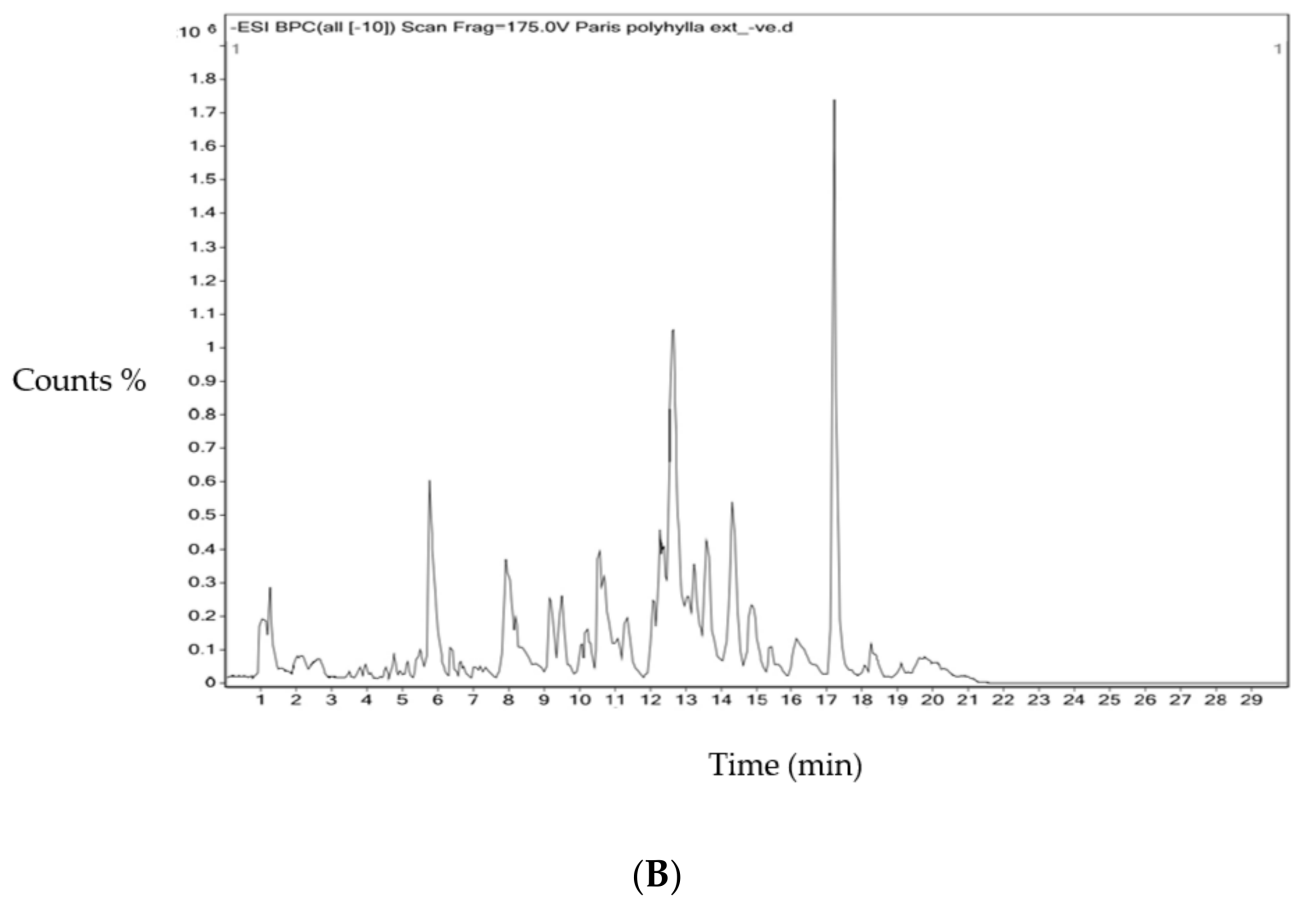
Total ion chromatogram of PPE: (**A**) Positive mode and (**B**) Negative mode of ionization.

PPE was subjected to HR-LCMS to identify the phytochemicals responsible for the anticancer activity. Compounds detected in the positive mode with anticancer properties were Paris saponin II [39], Polyphyllin III [40], Polyphyllin I [41], and Pennogenin [42] (Table 4) (Figure 9A). Polyphyllin G [43], Diosgenin [44], Polyphyllin E [45], Polyphyllin III [40], Polyphyllin I [46], and Polyphyllin VI [13] were detected in the negative mode (Table 5) (Figure 9B). Polyphyllin III and Polyphyllin I were detected in both positive and negative modes. The total compounds detected in the positive and negative modes are listed in Table S1 and Table S2.

## 3. Discussion

*P.polyphylla* is used all over the globe for its valuable health benefits through various traditional medicine systems [22]. The present study has revealed the role of *P. polyphylla* as a convincing plant for enhancing CRC therapy. PPE induces ROS production and apoptosis and potentiates the activity of 5FU and cisplatin in HCT-116.

The yield of 20 g dried rhizome of *P. polyphylla* extracted with ethanol–water (7:3, *v*/*v*) at room temperature was 4.6%, which is lower than that reported by Wu et al. [47], which was 1.5 kg (15%) with the same extraction method from dried rhizome of *P. polyphylla* var. *yunnanensis* (10 kg). The yields of *P. polyphylla* Sm. extract obtained from methanol (65 °C) and water (100 °C) extraction in Soxhlet apparatus were 8.9 % and 6.5%, respectively [48]. Extraction of *P. polyphylla* var. *chinensis* (2 kg) with hot methanol yielded 702 g (35%) [49]. In addition, 343 g (11.4%) of *P. polyphylla* extract was obtained from extraction with methanol three times at room temperature [20].

Phenols and flavonoids are secondary metabolites essential for plants with free hydroxyl groups and are accountable for anti-oxidant activity [50,51]. PPE’s TPC and TFC content was 34.4 mg GAE/g and 41.6 mg QE/g, respectively. The TPC and TFC of *P. polyphylla* from Manipur were higher than those reported in Sikkim, Himalaya [52]. PPE hada total saponin of 270.92 ± 0.19 mg DE/gand steroidal saponin content of 14.5 ± 0.02 mg DE/g. The anti-oxidant assays revealed that PPE showed dose-dependent free-radical-scavenging activity. PPE possesses potent anti-oxidant activity with IC_50_ 35.12 ± 6.1 μg/mL in DDPH and 19.69 ± 6.7 μg/mL in ABTS assays. An extract exhibits intense anti-oxidant activity if IC_50_ is 10–50 μg/mL [53]. Colorectal cancer is the third leading cause of cancer death worldwide and the most frequent cancer in incidence and mortality. Colorectal cancer cases also have a lower response rate due to various drug-resistance problems. The above problem limits the effectiveness of chemotherapy treatments. Multidrug resistance and significant dose-related toxicity restrict the practical application of chemotherapy [54]. *P. polyphylla* is a potent herb for cancer therapy, which can be exploited to produce natural cancer treatments since its phyto-compounds, such as steroidal saponins and triterpenoid saponins, have fewer side effects in humans than do synthetic drugs [22]. In our study, PPE is cytotoxic to HT-29, HCT-15, and HCT-116 in a dose-dependent manner, in which the most significant activity was observed in HCT-116 with the lowest IC_50_, 8.72 μg/mL. The viability of the normal human colon epithelial cell line, CCD 841 CoN, upon treatment with PPE was comparatively higher compared with the cancer cell lines, showing that PPE is less toxic to the normal colon cell line. Inhibition of colony and migration in bladder cancer cell line J82 after treatment with *P. polyphylla* ethanol extract has been reported [55]. Paris saponin II and PP9 inhibit colony formation in HT-29 and HCT-116 cell lines [56,57]. *P. polyphylla* fruit extract inhibits colorectal cancer (Caco-2) cell migration [58]. Similarly, clonogenic and scratch wound assay in our study revealed a significant inhibition potential of PPE on the invasive capability of HCT-116 cells in a dose- and time-dependent manner. Our result shows that PPE restrains the properties of cancer cells (HCT-116) that lead to metastasis. Intracellular baseline ROS concentration plays a crucial role in controlling signaling cascades, while at a higher concentration, it triggers apoptosis [59]. ROS regulates certain enzymes in the cell death pathway during apoptosis [60]. DLD-1 and HCT-116 cells exposed to pennogenin 3-*O*-beta-chacotrioside, Polyphyllin VI, and Polyphyllin I isolated from *P. polyphylla* ethanolic extract trigger ROS-induced autophagy [28,41]. It has also been reported that Polyphyllin VII from *P. polyphylla* induces ROS-mediated apoptosis in HepG2 cells by activating MAPK (*mitogen-activated protein kinase)*, PTEN (phosphatase and tensin homolog), and p53 pathways [61]. Polyphyllin I increases the production of ROS and inhibits the AKT/mTOR pathway, which encourages colon cancer cells to undergo apoptosis and autophagic cell death [62]. In the present study, PPE stimulated ROS production and externalized PS in Annexin V/PI staining assay, confirming the induction of ROS-mediated apoptosis in HCT-116. Although the anti-oxidant activity of phytochemicals is well recognized, they can also display prooxidant activities under certain conditions, such as at high doses or in the presence of metal ions [63]. PPE could act as a facilitator for intracellular reactive oxygen-scavenging system at very low doses (5, 10, and 15 μg/mL) and act as a prooxidant at higher doses (25, 50, and 100 μg/mL) in HCT-116. Similarly, ascorbic acid scavenges intracellular ROS at low doses (20 μg/mL and 50 μg/mL) and increases intracellular ROS at a high dose (200 μg/mL). Our result is consistent with previous report of ascorbic acid, showing both anti-oxidant and prooxidant at low and high doses, respectively [64,65]. Cell apoptosis is initiated by caspase 3, an enzyme belonging to the cysteine protease family. In viable cells, caspase 3 is in an inactive procaspase activated during apoptosis, resulting in the cell’s death [66]. PPE activated caspase 3 in HCT-116 in a dose-dependent manner, ultimately leading to apoptosis. The synergic effect of *P. polyphylla* extract with 5FU and Oxaplatin in the human hepatoma cell line has been reported [67]. *P. polyphylla 26*(PP-26) purified from *P. polyphylla* infused with 5FU enhanced the inhibition of HepG2 [68]. These findings are on par with our result, where the synchronous treatment of PPE increased the sensitivity of the chemotherapeutic drugs significantly. PPE (2, 3, 5, and 7 μg/mL) acts synergistically with 5FU (130 μg/mL). Similarly, PPE (3, 5, and 7 μg/mL) with cisplatin (300 μg/mL) showed synergistic combination in HCT-116. Our outcome on the activity of PPE in imparting anticancer activity in HCT-116 is attributed to the steroidal saponins Paris saponin II, Polyphyllin III, Polyphyllin I, Pennogenin, Polyphyllin G, Diosgenin, Polyphyllin E, and Polyhpyllin VI detected in PPE by HR-LCMS.

The present study showed that PPE could be a potential natural remedy for CRC treatment. PPE could be used in conjunction with 5FU or cisplatin in low doses to enhance the treatment and reduce the side effects. PPE showed similar activity comparable to 5FU at very low doses. The difference in the quantity of secondary metabolites produced by plants depends on the geographical location, environment, time of harvest, etc. [69]. Further comparison of the phytochemical content of *P. polyphylla* from various geographical places is required to comprehend the quality of the variety found in Manipur. To validate the anticancer activity, further studies are needed to isolate and elucidate the bioactive compounds responsible for this activity and to clarify the underlying molecular pathway.

## 4. Materials and Method

### 4.1. Chemical Reagents

Ethanol, sodium carbonate (99.9%), potassium persulfate (99%), sulphuric acid (95–97%), crystal violet (99.7%), and DPPH (98.9%) were obtained from Himedia (Thane (MH), India). Folin-Ciocalteu, Hydrogen peroxide (30%) was purchased from Merk (Khalapur (MH), India). L-Ascorbic acid (99%), Gallic acid (≥98%), Quercetin (≥99%), Diosgenin (≥93%), Vanillin (≥97%), 5FU (≥99%), and Cisplatin (≥98%) were obtained from Sigma (St. Louis (MO), USA) and ABTS (>98%) from Sigma (Oakville, (ON), Canada). Aluminium chloride (98%) was supplied by Finar (Ahmedabad (GJ), India). Anisaldehyde was obtained from Central drug house (CND) (Darya Ganj (ND), India). DMSO, ethyl acetate, and methanol were obtained from Rankem (Gurgaon (HR), India). DMEM, Pen Strep, PBS, Trypsin and FBS from Gibco (Paisley (SCT), UK), and Whatman filter paper, MTT (≥98%), H_2_DCFDA (≥97%), Annexin V/PI, and EnzChek^®^ Caspase-3 Assay Kit #1were purchased from Thermofisher Scientific (Waltham (MA), USA).

### 4.2. Plant Material

*P. polyphylla* is known as love apple in English, locally known as Singpan in Manipur and Kazeapai by the Tangkhul tribe in the Ukhrul district. The rhizome of *P. polyphylla* is a vital ingredient used in several patented Traditional Chinese herbal formulations; in this regard, we used the rhizome for our study. The rhizome was collected in January 2019 from Ukhrul, a hill station located in the northern part of Manipur from an altitude of 1811 m above sea level (latitude: N 25°06′42.3″, longitude: E 094°24′28.7″). The plant was identified and authenticated as *P. polyphylla* Sm. (Melanthiaceae) by the Botanical Survey of India (BSI), Central national herbarium Howrah-711103 (Specimen No. IBSD/KV-01).

### 4.3. Extraction

A critical literature search revealed the use of the hydroalcoholic solution as a solvent for extracting *P. polyphylla* rhizome, which induces anticancer activity [17,44,55]. *P. polyphylla* rhizome was washed in running water, cut into small pieces, and shade-dried for a week. The dried sample was then ground into fine powder form.Then, 20 g of *P. polyphylla* powdered sample was extracted by cold maceration using 100 mL hydroalcoholic solution (ethanol–water, 7:3 *v*/*v*) for 72 h at room temperature. The extract was then filtered through Whatman filter paper (No. 4, pore size of 20–25 µm), and the filtrate was concentrated to dryness using a rotary vacuum evaporator (IKA RV 10). The thick paste *P. polyphylla* extract (PPE) obtained was then stored at −20 °C for further use.

### 4.4. Yield of the Extract

The overall yield of the PPE extract was calculated using the formula [70]

(1) $$\%\;\mathrm{Yield}=\frac{\mathrm{Weight}\;\mathrm{of}\;\mathrm{the}\;\mathrm{evaporated}\;\mathrm{PPE}}{\;\mathrm{Dry}\;\mathrm{weight}\;\mathrm{of}\;\mathrm{PPE}}\times 100$$

### 4.5. Phenol and Flavonoid Quantification

#### 4.5.1. Total Phenolic Content (TPC)

The total phenolic content (TPC) in PPE was evaluated by the Folin-Ciocalteu method [71] with slight modifications. PPE and gallic acid of concentration 1 mg/mL were prepared in methanol, and 500 μL of PPE/gallic acid was added in a test tube, followed by 2.5 mL of Folin-Ciocalteu reagent and 2 mL of 7.5% (*w*/*v*) sodium carbonate. Reagent blank without Folin-Ciocalteu reagent and control without sample/standard wereadded. The mixture was allowed to stand for 30 min at room temperature, and the absorbance was measured spectrophotometrically at 760 nm (Varioskan Lux). The TPC was represented as the mean of three experiments in milligrams of gallic acid equivalent (GAE) per gram dry weight of PPE (mg GAE/g).

#### 4.5.2. Total Flavonoid Content (TFC)

The aluminium chloride colourimetric assay was used to determine PPE total flavonoid content (TFC) [72]. The amount of 1 mL of PPE/Quercetin (1mg/mL) prepared in methanol and an equal volume of aluminium chloride (10%) solution prepared in methanol weretaken in a test tube and incubated for 30 min at room temperature. Reagent blank without aluminium chloride and control without sample/standard wereadded. After that, the absorbance of the mixture was measured spectrophotometrically at 415 nm (Varioskan Lux). The TFC was expressed as the mean of three experiments in milligrams of quercetin equivalent (QE) per gram dry weight of PPE (mg QE/g).

### 4.6. Steroidal Saponin Quantification

#### 4.6.1. Total Saponin Content (TSC)

Total saponin content was determined spectrophotometrically [73]. To a test tube was added 250 μL of PPE extract/Diosgenin 1mg/mL in methanol, followed by 250 μL 8% vanillin reagent prepared in absolute ethanol and 2.5 mL of 72% sulphuric acid *v*/*v*. The tube was allowed to react at 60 °C in a water bath for 10 min. Reagent blank without vanillin reagent and control without sample/standard wereadded. After cooling, the absorbance of the mixture was measured at 544 nm using a spectrophotometer (Varioskan Lux). TSC was expressed as the mean of three experiments in milligrams of Diosgenin equivalent (DE) per gram dry weight of PPE (mg DE/g).

#### 4.6.2. Total Steroidal Saponin Content (TSSC)

Total steroidal saponin content was determined by Baccou et al. [74]. The amount of 1 mg/mL of PPE/Diosgenin was prepared in ethyl acetate for estimation. In a test tube, 100 μL of PPE/standard Diosgenin wasadded, followed by 1.9 mL of ethyl acetate, 1 mL of (0.5:95.5, *v*/*v*) anisaldehyde and ethyl acetate, and 1 mL of (50:50, *v*/*v*) sulphuric acid (95–97%) and ethyl acetate. A control without sample/standard was added. The mixture was vortexed and reacted at 60 °C for 10 min. After cooling, the absorbance of the mixture was measured at 430 nm using a spectrophotometer (Varioskan Lux). TSSC was expressed as the mean of three experiments in milligrams of Diosgenin equivalent (DE) per gram dry weight of PPE (mg DE/g).

### 4.7. Anti-Oxidant Assays

#### 4.7.1. DPPH (2,2-Diphenyl-1-picrylhydrazyl) Radical Scavenging Assay

The free-radical-scavenging activity of PPE was determined by DPPH as described previously [75] with slight modifications. To a 96-well plate were added 100 μL of freshly prepared 0.1 mM DPPH in 90% methanol and 100 μL of PPE (5–150 μg/mL)/ ascorbic acid (5–150 μg/mL) prepared in distilled. Reagent blank without DPPH and control without sample/standard were added. The mixture was incubated for 30 min in the dark at room temperature, and the absorbance was measured spectrophotometrically at 517 nm (Varioskan Lux). The presence of anti-oxidants will change the violet/purple colour of the DPPH solution to faint yellow. The DPPH scavenging effect was measured using the following formula:

(2) $$\%\;\mathrm{DPPH}\;\mathrm{radical}\;\mathrm{scavenging}\;\mathrm{activity}=\frac{A0-A1}{A0}\times 100$$

where A0 = absorbance of the control, and A1= absorbance of the extract/standard.

The concentration of PPE required to scavenge 50% of the DPPH free radical (IC_50_) was calculated using GraphPad Prism 8.4.3, San Diego, CA, USA.

#### 4.7.2. ABTS (2,2′-Azino-bis-(3-ethylbenzothiazoline-6-sulfonic acid)

PPE’s free-radical-scavenging activity was also determined by the ABTS radical cation decolourization assay [76,77], in which the blue/green ABTS is reduced to colourless in the presence of anti-oxidants. The amount of 7 mM ABTS was prepared in deionized water, and ABTS radical cation was produced by mixing equal volume with 2.45 mM potassium persulfate, and the mixture was allowed to stand in the dark for 12–16 h. Before the experiment, the ABTS+ solution was diluted in deionized water to obtain an absorbance of 0.7 ± 0.02 at 734 nm. In a 96-well plate, 100 μL of PPE (5–150 μg/mL)/ ascorbic acid (5–150 μg/mL) prepared in distilled water was mixed with 100 μL ABTS+ solution and incubated at room temperature for 7 min. Reagent blank without ABTS and control without sample/standard wereadded. After incubation, the absorbance was measured at 734 nm in a spectrophotometer (Varioskan Lux). The ABTS scavenging effect was measured using the following formula:

(3) $$\%\;\mathrm{ABTS}\;\mathrm{scavenging}\;\mathrm{activity}\;=\frac{A0-A1}{A0}\times 100$$

where A0 = absorbance of the control, and A1 = absorbance of the extract/standard. The concentration of PPE required to scavenge 50% of the ABTS free radical (IC_50_) was calculated using GraphPad Prism 8.4.3 San Diego, CA, USA.

### 4.8. Cell Culture

The human CRC cell lines HT-29, HCT-15, and HCT-116 were used for our study, as these cells are used in numerous biomedical studies for investigating colon cancer proliferation and the effectiveness of corresponding inhibitors. HT-29, HCT-15, and HCT-116 were obtained from the National Centre for Cell Science (NCCS), Pune, India, and cultured in DMEM (Dulbecco’s Modified Eagle Medium) supplemented with 10% FBS (Fetal Bovine Serum) and 1% Pen Strep maintained at 37 °C in 5% CO_2_. Normal human colon epithelial cell line, CCD 841 CoN, was kindly gifted by Dr. VGM Naidu (National Institute of Pharmaceutical Education and Research, Guwahati) and cultured in MEM (Minimum Essential Media) supplemented with 10% FBS (Fetal Bovine Serum) and 1% Pen Strep maintained at 37 °C in 5% CO_2._

#### 4.8.1. MTT (3-(4,5-dimethylthiazol-2-yl)-2,5-Diphenyltetrazolium bromide) Assay

MTT assay was used to determine the viability of the colon cancer cells treated with PPE and compared with normal human colon epithelial cell line, CCD 841 CoN. Viable cells convert MTT (3-(4,5-dimethylthiazol-2-yl)-2,5-diphenyl tetrazolium bromide) to purple formazan. The quantity of live cells directly relates to the intensity of the purple formazan [78]. HT-29, HCT-15, HCT-116, and CCD 841 CoN cells were seeded in a 96-well plate with 5 × 10^3^ cells in 100 μL media and allowed to adhere for 24 h. Subsequently, the cells were treated with PPE (1–100 μg/mL prepared in milli-Q water) and 5FU (0.03–1.5 mM dissolved in DMSO with final DMSO concentration <0.1%) for 24 h. Untreated cells with media only were used as control. Thereafter, 15 μL of MTT (5 mg/mL) prepared in distilled water was added to all the wells and incubated for 4 h. After incubation, the medium was removed, and the formazan formed was dissolved in 100 μL DMSO. A reading of the optical density of the plate was taken at 570 nm in a microplate reader (Varioskan Lux). The IC_50_ values (half maximal inhibitory concentration) required to inhibit the cell lines were calculated using GraphPad Prism 8.4.3 San Diego, California.

#### 4.8.2. Clonogenic Assay

The clonogenic or colony formation assay is an in vitro assay in which the ability of a single cell in its exponential growth phase to proliferate into colonies is determined [79]. HCT-116 cells were seeded in 6-well plates with a cell density of 600–700 cells per well and incubated overnight. The medium was removed, and the cells were treated with PPE (3 μg/mL, 5 μg/mL, and 10 μg/mL prepared in milli-Q water) and 5FU (1.1 mM dissolved in DMSO with a final concentration of <0.1%). Untreated cells with media only were used as control. After 24 h, the cells were washed with 1XPBS to remove the PPE and replaced with a fresh culture medium. The colony-forming ability of the cells was observed for 10 days. The colonies were counted manually after staining with crystal violet (0.5% *w*/*v* in methanol).

#### 4.8.3. Cell Migration Assay

Cell migration plays a vital role in the invasion and metastasis of cancer. The role of PPE in HCT-116 cell migration was determined using invitro scratch wound assay [80]. HCT-116 cells were seeded in a 6-well plate to reach 80–90% confluence. With a sterile 200 μL pipette tip, a wound was introduced to the monolayer of cells. The cells were washed three times with 1XPBS and treated with PPE (2 μg/mL and 3 μg/mL, prepared in milli-Q water) and 5FU (1.1 mM dissolved in DMSO with a final concentration of <0.1%).Untreated cells with media only wereused as control. The scratched monolayer of cells without treatment was considered a control. Images of the wound closure were captured at 0 h, 24 h, and 48 h. The closure length of the scratched wound was measured by ImageJ 1.53 e software, Bethesda, MD, USA.

#### 4.8.4. Measurement of Reactive Oxygen Species (ROS)

ROS build-up in cells causes damage to proteins, nucleic acids, lipids, membranes, and organelles, resulting in the activation of cell death processes, such as apoptosis. ROS are involved in significant aspects of cell signaling and in the main pathways involved in apoptosis, such as mitochondrial function and death receptors [81]. The intracellular ROS generation upon treatment with PPE was measured with 2′,7′-dichlorodihydrofluorescein di-acetate (H_2_DCFDA). In the presence of ROS, non-fluorescent H_2_DCFDA is converted to highly fluorescent 2′,7′-dichlorofluorescein (DCF). HCT-116 cells were seeded in a 6-well plate and treated with high and low concentrations of PPE (5, 10, 15, 25, 50, and 100 μg/mL prepared in milli-Q water) andhydrogen peroxide (H_2_O_2_) (500 μM) for 6 h, as high doses of PPE induces cell death in 24 h, and ascorbic acid (AA) (20, 50 and 200 μg/mL prepared in milli-Q water) for 24 h. Untreated cells with media only wereused as control. Thereafter, the cells were collected by trypsinization and resuspended in 1XPBS. The cells were incubated with 5 μM H_2_DCFDA in the dark for 30 min [82], and the cells were analyzed by flow cytometer (BD Canto-II).

#### 4.8.5. Annexin V/PI (Propidium Iodide) Staining

The morphological and biochemical changes associated with apoptosis, characterized by nuclear chromatin fragmentation, cytoplasm shrinkage, and loss of membrane symmetry distinguish it from necrosis or accidental cell death. Annexin V binds to phosphatidyl serine (PS), which translocates to the outer plasma membrane in apoptotic cells, and PI stains dead or necrotic cells [83]. HCT-116 cells were seeded in 6-well plates and treated with PPE (5–15 μg/mL prepared in distilled water) and 5FU (1.1 mM dissolved in DMSO with a final concentration of <0.1%) for 24 h. Untreated cells with media only wereused as control. After that, the cells were harvested by trypsinization and washed with ice-cold 1XPBS. The cells were processed according to the manufacturer’s protocol (Invitrogen FITC Annexin V/Dead Cell Apoptosis Kit with FITC annexin V and PI, for Flow Cytometry) and analyzed by flow cytometer (BD Canto-II). Post-acquisition analysis was conductedusing FCS Express 7.

#### 4.8.6. Caspase 3 Activity Assay

Caspase 3’s activation with substrate-specific amino acids Asp-Glu-Val-Asp (DEVD) is an essential mediator of apoptosis. Caspase 3 is a death protease frequently activated during apoptosis [66]. Activation of Caspase 3 in HCT-116 upon treatment with PPE was evaluated by using EnzChek^®^ Caspase-3 Assay Kit #1, Thermofisher Scientific Waltham (MA), USA. HCT-116 cells were seeded in a 6-well plate and treated with PPE (5–15 μg/mL prepared in distilled water) and 5FU (1.1 mM dissolved in DMSO with a final concentration of <0.1%) for 24 h. Untreated cells with media only wereused as control. After that, the cells were harvested and washed with ice-cold 1XPBS. The cells were processed according to the manufacturer’s protocol. The fluorescent intensity of 7-amino-4-methylcoumarin-derived substrate Z-DEVD–AMC (excitation/emission ~342/441 nm) upon proteolytic cleavage by active caspase 3 was measured at an incubation time of 30, 60, and 120 min. The fluorescence intensity is proportional to caspase 3 activity. The amount of AMC released in the reaction was quantified using the reference standard, 7AMC, for an incubation time of 120 min.

#### 4.8.7. Combinational Evaluation of Cisplatin and 5FU with PPE in HCT-116

To determine the combinational treatment of cisplatin, 5FU, and PPE, HCT-116 cells were seeded in a 96-well plate with cell density 5 × 10^3^ in 100 μL media and incubated overnight. After that, the cells were treated with a sub-lethal dose of 5FU (16, 32, 65, 95, and 130 μg/mL dissolved in DMSO with a final concentration of <0.1%) or cisplatin (27, 32, 75, 150, and 300 μg/mL dissolved in DMSO with a final concentration of <0.1%) used in combination with a sub-lethal dose of PPE below IC_50_ (2,3,5, and 7 μg/mL) for 24 h. Untreated cells with media only wereused as control. The combinational effect was assessed by MTT assay using the same method described above.

The combinational effect of PPE with cisplatin and 5FU was calculated [84,85].

CI =C_A,*x*_/IC_𝑥,A_+ C_B,𝑥_/IC_𝑥,B_ (4)

where C_A,*x*_ = sub-lethal concentration of cisplatin or 5FU used in combination with PPE, C_B,*x*_ = sub-lethal concentration of PPE used in combination, IC*_x_*_,A_ = concentration of cisplatin or 5FU alone to achieve the same effect as the combination, and IC*_x_*_,B_ = concentration of PPE alone to achieve the same effect as the combination.

The value of the combination index (CI) determines the nature of the combinational effect as synergistic if CI < 1, additive if CI = 1, and antagonist if CI > 1. A heatmap for predicted drug combination effects of PPE with 5FU and cisplatin was generated using Microsoft Excel.

### 4.9. HRLCMS-ESI-QTOF-MS/MS

1290 Infinity UHPLC System, 1260 infinity Nano HPLC with Chipcube, and 6550 iFunnel Q-TOFs (Agilent Technologies, Santa Clara (CA), USA) were used to examine the phytochemicals in PPE. A Zorbax Eclipse Plus c18 RRHD column with an inner diameter of 2.1 mm and length of 100mm with 1.8 μm particle size (Agilent Technologies, Santa Clara (CA), USA) was used for the chromatographic separation of all metabolites under the following conditions: 45 °C temperature, injection volume of 10 μL (PPE of 10 ppm prepared in milli-Q water), and constant flow rate of 0.300 mL/min. The mobile phase consisted of (A) 0.1% formic acid in water and (B) 0.1% formic acid in acetonitrile, and metabolite separation was accomplished using a gradient method. The gradient approach was 0–20 min 95% A, 20–30 min 100% B, 30–31 min 95% A, and 31–35 min 100% A. The following parameters were optimized for the usual operating source circumstances for MS and MS/MS scans gas source temperature: 250°C; drying gas rate: 13 L/min; nebulizer pressure: 35 psig; spray voltage: ±3500 V; and cone voltage and sheath gas temperature: ±1000 V and 350 °C, respectively. Positive and negative ionization modes with *m*/*z* ranges of 120 to 1200 were used for MS and MS/MS studies. In both positive and negative modes, scan rates of 1.0 scans per second were used for the data capture of MS and MS/MS.

### 4.10. Statistical Analysis

The raw data obtained were analyzed using GraphPad Prism 8.4.3. We reported the results as mean ± SD. The IC_50_ was also calculated using GraphPad prism and represented in the graph with one-way analysis of variance (ANOVA), with *p* ≤ 0.05 considered to be statistically significant.

## 5. Conclusions

In summary, *P. polyphylla* from Manipur has high TPC, TFC, TS, and TSS content and has potent anti-oxidant activity. PPE induced apoptosis in HCT-116 by increasing ROS production and caspase 3 activations. PPE showed both prooxidant and anti-oxidant properties. Steroidal saponins with anticancer activities were detected in PPE by HR-LCMS. PPE acts synergistically with 5FU and cisplatin and potentiates their therapeutic properties.

## Figures and Tables

**Table 1:** IC_50_ values of ascorbic acid and PPE for their anti-oxidant activity.

Anti-Radical Assay	Ascorbic Acid (IC_50_)	PPE (IC_50_)
DPPH	14.9 ± 0.24 μg/mL	35.12 ± 6.1 μg/mL
ABTS	16.96 ± 0.4 μg/mL	19.69 ± 6.7 μg/mL

Values are mean ± SD, *n* = 3.

**Table 2:** IC_50_ values of PPE in CRC cells and CCD 841 CoN.

Cell Line	IC_50_ (μg/mL)
HCT-116	8.72 ± 0.71
HCT-15	10 ± 0.5
HT-29	12.94 ± 1.8
CCD 841 CoN	113.33 ± 1.65

Values are mean ± SD, *n* = 3.

**Table 3:** Combinational indices of 5FU and cisplatin with PPE at different doses, % cell viability, and the subsequent effects on HCT-116.

Combination (μg/mL)	% Cell Viability (Mean)	Combination Index (CI)	Effect
5FU 16 + 2 PPE	79	2.2	Antagonist
5FU 32 + 2 PPE	73	1	Additive
5FU 65 + 2 PPE	68	1	Additive
5FU 95+ 2 PPE	56	0.4	Synergistic
5FU 130 + 2 PPE	42	0.7	Synergistic
5FU 16 + 3 PPE	75	1.8	Antagonist
5FU 32 + 3 PPE	45	0.4	Synergistic
5FU 65 + 3 PPE	41	0.4	Synergistic
5FU 95+ 3 PPE	30	2.4	Antagonist
5FU 130 + 3 PPE	36	0.6	Synergistic
5FU 16 + 5 PPE	71	1.9	Antagonist
5FU 32 + 5 PPE	52	0.8	Synergistic
5FU 65 + 5 PPE	50	0.9	Synergistic
5FU 95+ 5 PPE	43	1	Additive
5FU 130 + 5 PPE	34	0.8	Synergistic
5FU 16 + 7 PPE	68	2	Antagonist
5FU 32 + 7 PPE	54	1.1	Antagonist
5FU 65 + 7 PPE	45	1	Additive
5FU 95+ 7 PPE	33	0.8	Synergistic
5FU 130 + 7 PPE	26	0.8	Synergistic
Cis 27 + 2 PPE	89	17	Antagonist
Cis 32 + 2 PPE	77	21.1	Antagonist
Cis 75 + 2 PPE	65	6.1	Antagonist
Cis 150 + 2 PPE	58	2.3	Antagonist
Cis 300 + 2 PPE	52	2.5	Antagonist
Cis 27 + 3 PPE	78	21.2	Antagonist
Cis 32 + 3 PPE	63	0.6	Synergistic
Cis 75 + 3 PPE	64	0.8	Synergistic
Cis 150 + 3 PPE	45	0.5	Synergistic
Cis 300 + 3 PPE	8	0.3	Synergistic
Cis 27 + 5 PPE	70	3.3	Antagonist
Cis 32 + 5 PPE	65	3.5	Antagonist
Cis 75 + 5 PPE	50	1	Additive
Cis 150 + 5 PPE	40	0.9	Synergistic
Cis 300 + 5 PPE	29	0.9	Synergistic
Cis 27 + 7 PPE	64	2.1	Antagonist
Cis 32 + 7 PPE	55	1.3	Antagonist
Cis 75 + 7 PPE	40	0.8	Synergistic
Cis 150 + 7 PPE	19	0.5	Synergistic
Cis 300 + 7 PPE	9	0.3	Synergistic

The value of the combination index (CI) determines the nature of the combinational effect as synergistic if CI < 1, additive if CI = 1, and antagonist if CI > 1.

**Table 4:** Mass spectral characteristics and tentative identification of anticancer compounds present in PPEusing HR-LCMS in positive mode.

Name	Differential (ppm)	Formula	Mass	*m*/*z*	RT (min)
Paris saponin II	−3.87	C_51_H_82_O_20_	1014.536	1037.5272	12.418
Polyphyllin III	−2.05	C_45_ H_72_ O_16_	868.4803	869.4869	12.495
Polyphyllin I	−2.69	C_44_ H_70_ O_16_	854.4641	855.4713	12.788
Pennogenin	−6.29	C_27_ H_42_ O_4_	430.3056	431.3127	13.357

**Table 5:** Mass spectral characteristics and tentative identification of anticancer compounds present in PPE using HR-LCMS in negative mode.

Name	Differential (ppm)	Formula	Mass	*m*/*z*	RT (min)
Polyphyllin G	−4.16	C_51_H_84_O_22_	1048.5411	1083.5114	7.811
Diosgenin 3-[glucosyl-(1->4)-rhamnosyl-(1->4)-[rhamnosyl-(1->2)]-glucoside]	2.12	C_51_H_82_O_21_	1030.5327	1065.5023	10.525
Polyphyllin E	−1.82	C_51_H_82_ O_20_	1014.5381	1049.5084	12.32
Polyphyllin III	−2.41	C_45_H_72_ O_16_	868.4799	903.4499	12.42
Polyphyllin I	−1.12	C_44_H_70_ O_16_	854.4654	889.4352	12.76
Polyphyllin VI	−2.44	C_39_H_62_ O_13_	738.4172	737.4103	13.116

Note: Differential (ppm) refers to mass error, $\mathrm{Diff}\;\left(\mathrm{ppm}\right)=\frac{\mathrm{Measured}\;\mathrm{mass}-\mathrm{Theroretical}\;\mathrm{mass}}{\mathrm{Theoretical}\;\mathrm{mass}}\times 1,000,000$.

## Data Availability

Data are available within the manuscript.

## Supplementary Materials

The following supporting information can be downloaded at: https://www.mdpi.com/article/10.3390/plants12071446/s1, Table S1: Mass spectral characteristics and tentative identification of compounds present in PPE using HRLCMS in positive mode. Table S2: Mass spectral characteristics and tentative identification of compounds present in PPE using HRLCMS in negative mode.

## Abbreviations

5FU, Fluorouracil; AA, Ascorbic acid; ABTS, 2,2′-azino-bis-(3-ethylbenzothiazoline-6-sulfonic acid; AMC,7-amino-4-methylcoumarin;CI, Combination index; Cis, Cisplatin; CRC, Colorectal cancer; DCF, 2′,7′-dichlorofluorescein; DPPH, 2,2-diphenyl-1-picrylhydrazyl; DE, Diosgenin equivalent; DMEM, Dulbecco′s Modified Eagle Medium; DMSO, Dimethyl sulfoxide; FBS, Fetal Bovine Serum; FITC, Fluorescein isothiocyanate; GAE, Gallic acid equivalent; H_2_DCFDA,2′,7′-dichlorodihydrofluorescein di-acetate; HRLCMS-ESI-QTOF-MS/MS, High resolution liquid chromatography, electrospray ionization quadrupole time-of-flight mass spectrometry; HPLC, high performance liquid chromatography; H_2_O_2_, hydrogen peroxide; IBSD, Institute of Bioresources and Sustainable Development; MEM, Minimum essential media; MTT, 3-(4,5-dimethylthiazol-2-yl)-2,5-diphenyltetrazolium bromide; NCCS, National Centre for Cell Science; PI, Propidium iodide; PPE, *Paris polyphylla* extract; Pen Strep, Penicillin–Streptomycin; PBS, Phosphate buffer saline; PS, Phosphatidylserine, QE, Quercetin equivalent; ROS, Reactive oxygen species; RRHD, Rapid Resolution High Definition; SD, Standard deviation; TCM, Traditional Chinese medicine; TFC, Total flavonoid content; TPC, Total phenolic content; TSC, Total saponin content; TSSC, Total steroidal saponin content; UHPLC, Ultra-High-Performance Liquid Chromatography; Z-DEVD–AMC,7-amino-4-methylcoumarin-derived substrate; 2′,7′-dichlorodihydrofluorescein di-acetate.
