# Supplementary material for: Paris polyphylla Sm. Induces Reactive Oxygen Species and Caspase 3-Mediated Apoptosis in Colorectal Cancer Cells In Vitro and Potentiates the Therapeutic Significance of Fluorouracil and Cisplatin
Source: Plants (Basel). 2023 Mar 25;12(7):1446. doi: 10.3390/plants12071446 (PMC10097216; doi:10.3390/plants12071446)
Supplement: Supplementary file 1 [file plants-12-01446-s001.zip › plants-2110719-supplementary.pdf]

C

Table S1: Mass spectral characteristics and tentative identification of compounds present in PPE using HRLCMS in positive mode.

Name	Diff (ppm)	Formula	Mass	m/z	RT
D-Fucosamine	11.13	C6 H13 N O4	163.0826	164.0899	0.912
N6,N6-Dimethyladenosine	13.6	C12 H17 N5 O4	295.124	296.1312	0.917
N-(1-Deoxy-1-fructosyl)proline	9.9	C11 H19 N O7	277.1134	278.1207	0.964
4-Hydroxy-L-threonine	-2.02	C4 H9 N O4	135.0534	136.0612	1.423
Pregabalin	10.75	C8 H17 N O2	159.1242	160.1314	1.499
Fenapanil	19.29	C16 H19 N3	253.153	276.1421	1.551
Prolyl-Arginine	4.84	C11 H21 N5 O3	271.1631	294.1523	1.557
Pirbuterol	9.56	C12 H20 N2 O3	240.1451	241.1524	1.61
Ketotifen	-1.43	C19 H19 N O S	309.1192	310.1261	2.073
N-(1-Deoxy-1-fructosyl)phenylalanine	6.55	C15 H21 N O7	327.1297	328.1368	2.077
Larixinic Acid	7.13	C6 H6 O3	126.0308	127.038	2.385
6-Methylquinoline	7.7	C10 H9 N	143.0724	144.0797	4.322
1,4-Dimethylpyrrolo[1,2-a]pyrazine	-5.87	C9 H10 N2	146.0853	169.0744	4.834
2-amino-8-oxo-9,10-epoxy-decanoic acid	-1.29	C10 H17 N O4	215.116	238.1052	4.994
Cyometrinil	-6.13	C10 H7 N3 O	185.06	186.0676	5.373
1,25-Dihydroxyvitamin D3-26,23-lactone	5.25	C27 H40 O5	444.2852	445.2928	6.154
Panaxytriol	0.53	C17 H26 O3	278.188	301.1772	5.849
Ecdysterone	5.15	C27 H44 O7	480.3062	481.3136	5.958
26-Glucosyl-1,3,11,22-tetrahydroxyergosta-5,24-dien-26-oate	1566.98	C34 H54 O11	637.3679	660.3568	6.393
Moxidectin	3147.18	C37 H53 N O8	637.3712	660.3573	6.467
Dihydrocapsaicin	8.43	C18 H29 N O3	307.2122	308.2193	7.79
Melongoside K	1.12	C51 H82 O22	1046.5286	1047.5363	8.026
Polyphyllin VI	-3.56	C39 H62 O13	738.416	739.4238	8.106
Dihydrodeoxystreptomycin	-0.21	C21H41N7O11	567.2865	568.2936	8.91
Schidigeragenin C	7.01	C27 H42 O4	430.3053	431.3125	9.414
23-Acetoxysoladulcidine	-12.56	C29 H47 N O4	473.3565	496.3459	10.217
5-(10,13-Nonadecadienyl)-1,3-benzenediol	0.76	C25 H40 O2	372.3025	395.2918	10.909
Oxolucidine B	11.77	C30 H49 N3O2	438.3768	506.3662	10.926
4-Hydroxy-3,5,4'-trimethoxystilbene	0.05	C17 H18 O4	286.1205	309.1096	11.81
Lucidine B	12.47	C30 H49 N3 O	467.3817	490.371	12.151
Smilagenone	6.83	C27 H42 O3	414.3106	415.3179	12.282
Paris saponin II	-3.87	C51H82O20	1014.536	1037.5272	12.418
Polyphyllin III	-2.05	C45 H72 O16	868.4803	869.4869	12.495
5-(12-Nonadecenyl)-1,3-benzenediol	0.73	C25 H42 O2	374.3182	397.3075	12.587
Polyphyllin I	-2.69	C44 H70 O16	854.4641	855.4713	12.788
1-Monopalmitin	0.65	C19 H38 O4	330.2768	353.2659	12.943
Polyphyllin C	-3.28	C39 H62 O12	722.4218	723.4289	13.005
Pennogenin	-6.29	C27 H42 O4	430.3056	431.3127	13.357
Palmitic Acid	-1.66	C16 H32 O2	256.2407	279.23	13.495

Retapamulin	15.78	C30 H47 N O4 S	517.3144	518.3215	13.587
Citronellyl hexanoate	-0.08	C16 H30 O2	254.2246	277.2138	14.03
23-Acetoxysoladulcidine	5.28	C29 H47 N O4	473.348	496.3373	15.904
Mycinamicin VII	-19.13	C29 H47 N O7	521.3452	522.3525	16.493
Stearic acid	1.53	C18 H36 O2	284.2711	307.2602	16.566
2R-hydroxy-stearic acid	1.26	C18 H36 O3	300.266	323.2553	16.849

Table S2: Mass spectral characteristics and tentative identification of compounds present in PPE using HRLCMS in negative mode.

**Name**	**Diff (ppm)**	**Formula**	**Mass**	**m/z**	**RT**
Epidermin	3.44	C11H19NO6	261.1203	306.1185	2.668
Resorcinol	0.51	C6 H6 O2	110.0367	109.0296	3.565
Vanillic acid	4.18	C8 H8 O4	168.0416	167.0341	4.198
3,5-dihydroxybenzoic acid	4.69	C7 H6 O4	154.0259	153.0186	4.55
Caffeic acid	4.77	C9 H8 O4	180.0414	179.0343	4.684
1,8-Dihydroxy-3-hydroxymethylanthraquinone 1,8-di-O-b-D-glucoside	2.17	C27H30 O15	594.1572	593.1499	4.382
Kaempferol 3-rhamnoside 7-xyloside	2.1	C26H28 O14	564.1467	563.1391	5.375
m-Coumaric acid	5.06	C9 H8 O3	164.046	163.0393	5.458
6''-O-Acetylglycitin	1.81	C24H24 O11	488.131	533.1293	5.72
Ajugasterone C	-1.54	C27 H44 O7	480.3094	525.3081	5.786
Pisumionoside	0.96	C19 H32 O9	404.2042	449.2025	5.861
Isoferulic acid	5.58	C10 H10 O4	194.0568	193.0503	6.114
Polyphyllin G	-4.16	C51H84 O22	1048.5411	1083.5114	7.881
Isolobinine	7.11	C18H25NO2	287.1865	322.1558	8.884
Diosgenin 3-[glucosyl-(1->4)-rhamnosyl-(1->4)-[rhamnosyl-(1->2)]-glucoside]	2.12	C51H82 O21	1030.5327	1065.5023	10.525
1alpha,3beta,22R-Trihydroxyergosta-5,24E-dien-26-oic acid3-O-b-D-glucoside 26-O-[b-D-glucosyl-(1->2)-b-D-glucosyl] ester	1.8	C46H74 O20	946.4756	945.4686	10.61
Polyphyllin E	-1.82	C51H82 O20	1014.5381	1049.5084	12.32
Polyphyllin III	-2.41	C45H72 O16	868.4799	903.4499	12.42
Polyphyllin I	-1.12	C44H70 O16	854.4654	889.4352	12.76
Polyphyllin C	-1.74	C39H62 O12	722.4229	767.4212	12.98
Polyphyllin VI	-2.44	C39H62 O13	738.4172	737.4103	13.116
Gingerglycolipid B	1.06	C33H58 O14	678.3819	713.3515	13.375
Kolanone	-9.48	C33 H42 O4	502.3131	561.3271	14.161
Triterpenoid	12.07	C30 H48 O7 S	552.3054	551.2992	14.412
Tricornine	-3.65	C27 H43 N O8	509.3007	554.3009	14.89
Linalyl caprylate	1.99	C18 H32 O2	280.2397	279.2324	17.129
Malvalic acid	1.37	C18 H32 O2	280.2398	279.2326	17.194
Tetradecyl sulfate	1.69	C14 H30 O4 S	294.186	293.1787	19.736
